# A Complicated Multiloculated Pyogenic Liver Abscess With Spontaneous Rupture and Life-Threatening Hemorrhage Successfully Managed With Transarterial Embolization: A Case Report

**DOI:** 10.7759/cureus.108872

**Published:** 2026-05-15

**Authors:** Md Monirul Azim, Abdul Haleem Abbasi, Ameena Rahman

**Affiliations:** 1 Acute Medicine, Broomfield Hospital, Chelmsford, GBR; 2 General Internal Medicine, Broomfield Hospital, Chelmsford, GBR; 3 General Medicine, Broomfield Hospital, Chelmsford, GBR

**Keywords:** interventional radiology, polymicrobial infections, pyogenic liver abscesses, subcapsular hepatic hematoma, transarterial embolization

## Abstract

Pyogenic liver abscess is a severe intra-abdominal infection associated with significant morbidity and mortality, particularly in elderly and comorbid patients. While standard management involves antimicrobial therapy and percutaneous drainage, treatment failure can occur in the setting of multiloculated cavities or resistant polymicrobial infections. Spontaneous rupture of a liver abscess leading to a massive subcapsular hematoma and active hemorrhage is an exceedingly rare but potentially fatal complication. We present the case of an 83-year-old man who presented with sepsis secondary to a large, multiloculated right hepatic abscess. Despite initial broad-spectrum antibiotics and percutaneous drainage, the patient's condition deteriorated. Microbiological analysis, including 16S ribosomal RNA sequencing, revealed a complex polymicrobial infection comprising *Pseudomonas aeruginosa*, vancomycin-resistant *Enterococcus faecium*, and Candida species. His clinical course was acutely complicated by the spontaneous rupture of the abscess, resulting in a massive subcapsular hepatic hematoma, active arterial extravasation, and disseminated intravascular coagulation. The patient was successfully resuscitated and managed with urgent interventional radiology-guided transarterial embolization (TAE), which achieved rapid hemostasis and averted the need for high-risk surgical intervention. Following a prolonged multidisciplinary recovery complicated by antibiotic-related hepatotoxicity, he was successfully discharged. This case underscores the critical importance of early recognition of drainage failure, the utility of advanced microbiological diagnostics, and the life-saving role of TAE in managing hemorrhagic complications of hepatic abscesses.

## Introduction

Pyogenic liver abscess (PLA) is a severe, life-threatening intra-abdominal infection with reported mortality rates ranging from 2% to 15%, which can be significantly higher in elderly patients or those with multiple comorbidities [1]. The standard of care for PLA involves a combination of targeted antimicrobial therapy and image-guided percutaneous drainage [2]. However, treatment failure is well-documented, particularly in cases involving multiloculated abscesses, large cavity sizes, or infections driven by multidrug-resistant organisms [3]. While pleural eﬀusions and localized peritonitis are known complications, the spontaneous rupture of a PLA resulting in a massive subcapsular hepatic hematoma and active arterial hemorrhage is an exceedingly rare and catastrophic event [4]. Such hemorrhagic complications can rapidly precipitate hemorrhagic and septic shock, necessitating immediate intervention. We report a rare case of a multiloculated, polymicrobial PLA complicated by spontaneous rupture, massive subcapsular hematoma, and disseminated intravascular coagulation (DIC), which was successfully managed through urgent transarterial embolization (TAE) by interventional radiology (IR).

## Case presentation

An 83-year-old man presented to the emergency department with a history of acute confusion, reduced mobility, and persistent fever despite receiving outpatient oral antibiotics. His past medical history was signiﬁcant for atrial ﬁbrillation, aortic regurgitation, Barrett’s esophagus, a hiatus hernia, hypertension, and angina. Upon admission, he was tachycardic and hypotensive, consistent with sepsis. Initial laboratory investigations revealed markedly elevated inflammatory markers, including a C-reactive protein level greater than 260 mg/L and a white cell count of 14 × 10^9^/L (Table 1).

**Table 1:** Summary of important laboratory results APTT: activated partial thromboplastin time

Name of test	Patient value	Normal range
C-reactive protein	>260 mg/L	<5 mg/L
White cell count	14 × 10⁹/L	4-11 × 10⁹/L
Hemoglobin	Drop from 110 → 70 g/L	130-170 g/L (male)
Prothrombin time	47.2 seconds	11-13.5 seconds
International normalized ratio	3.92	0.8-1.2
APTT	44.9 seconds	25-35 seconds
APTT ratio	1.55	0.8-1.2
Alanine aminotransferase	130 U/L	7-56 U/L
Alkaline phosphatase	586 U/L	44-147 U/L
Total bilirubin	25 µmol/L	3-20 µmol/L

**Figure 1:**
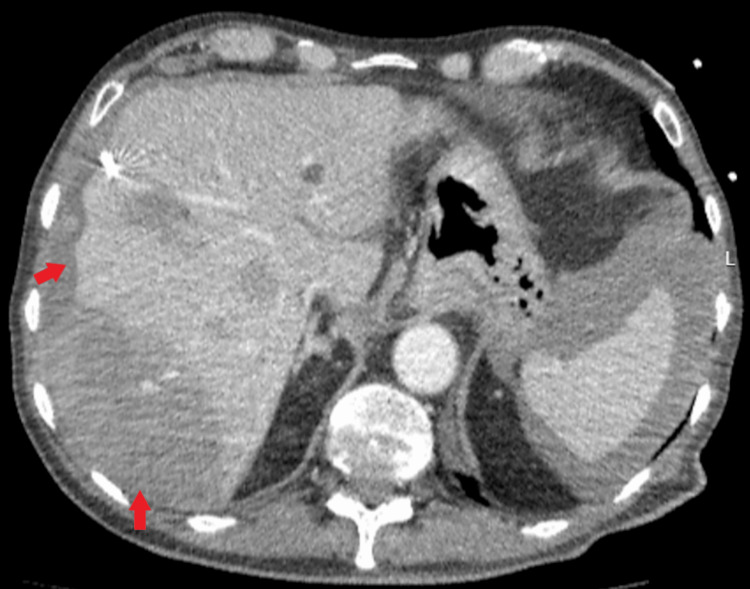
Contrast-enhanced CT abdomen and pelvis showing large right hepatic hematoma on Day 50 There is a large right-sided hepatic hematoma on this portal venous phase study CT: computed tomography

An urgent contrast-enhanced computed tomography (CT) scan of the thorax, abdomen, and pelvis was performed to identify the source of sepsis. The imaging demonstrated a large, complex, multiloculated hepatic abscess measuring approximately 10 cm in maximum diameter, located in the right hepatic lobe (Figure 1). Based on these findings, the patient commenced on intravenous piperacillin-tazobactam. Following multidisciplinary discussion with the microbiology team, his antimicrobial regimen was changed to intravenous ceftriaxone and metronidazole. Concurrently, IR was consulted, and the patient underwent successful image-guided percutaneous catheter drainage of the right hepatic abscess on Day 15.

**Figure 2:**
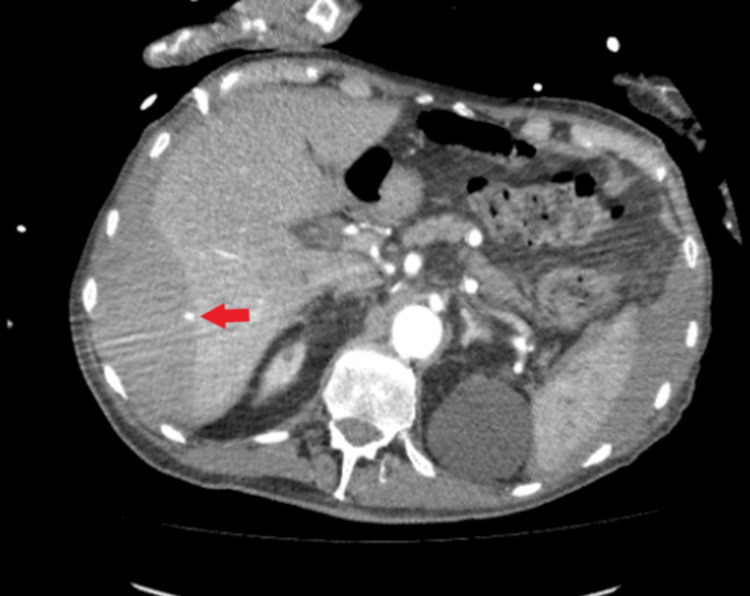
CT angiogram mesenteric artery on Day 50 showing large subcapsular hematoma with a focus of active bleeding There is a focus on arterial extravasation. There is evidence of mild contrast pooling in the venous phase CT: computed tomography

Despite initial drainage and broad-spectrum antibiotic therapy, serial follow-up CT imaging revealed an initial reduction in abscess size, followed by subsequent progression and the formation of further multiloculated collections. The patient's clinical condition acutely deteriorated, characterized by sudden hemodynamic instability and a precipitous drop in hemoglobin levels. An emergency CT angiogram was performed, which revealed the spontaneous rupture of the hepatic abscess complicated by a massive subcapsular hepatic hematoma (Figure 2). Crucially, arterial-phase imaging demonstrated active contrast extravasation within the hematoma, indicating ongoing arterial hemorrhage.

Given the patient's advanced age, comorbidities, and hemodynamic instability, he was deemed a poor candidate for open surgical intervention. He was urgently transferred to the IR suite for TAE. Selective superior mesenteric artery/hepatic artery accessed with a 5-Fr C2 catheter. Subselective hepatic artery cannulation was performed with a progressive microcatheter. The bleeding point was identified inferiorly in the right lobe of the liver. Feeding vessel embolized with 2/3 and 3/4 mm tornado coils. Bleeding stopped after the hemorrhage.

The patient's clinical course was further complicated by the development of DIC secondary to the profound septic and hemorrhagic shock, which was managed with aggressive supportive care, including the administration of blood products and fresh frozen plasma. Microbiological analysis of the drained abscess ﬂuid, enhanced by 16S ribosomal RNA (rRNA) sequencing, revealed a polymicrobial infection. The cultures yielded heavy growth of *Pseudomonas aeruginosa*, vancomycin-resistant *Enterococcus faecium* (VRE), and Candida species. Guided by these sensitivities, the antimicrobial regimen was escalated to include meropenem, daptomycin, levoﬂoxacin, and tigecycline. However, tigecycline was subsequently discontinued due to the development of drug-induced hepatotoxicity, a known adverse effect.

**Figure 3:**
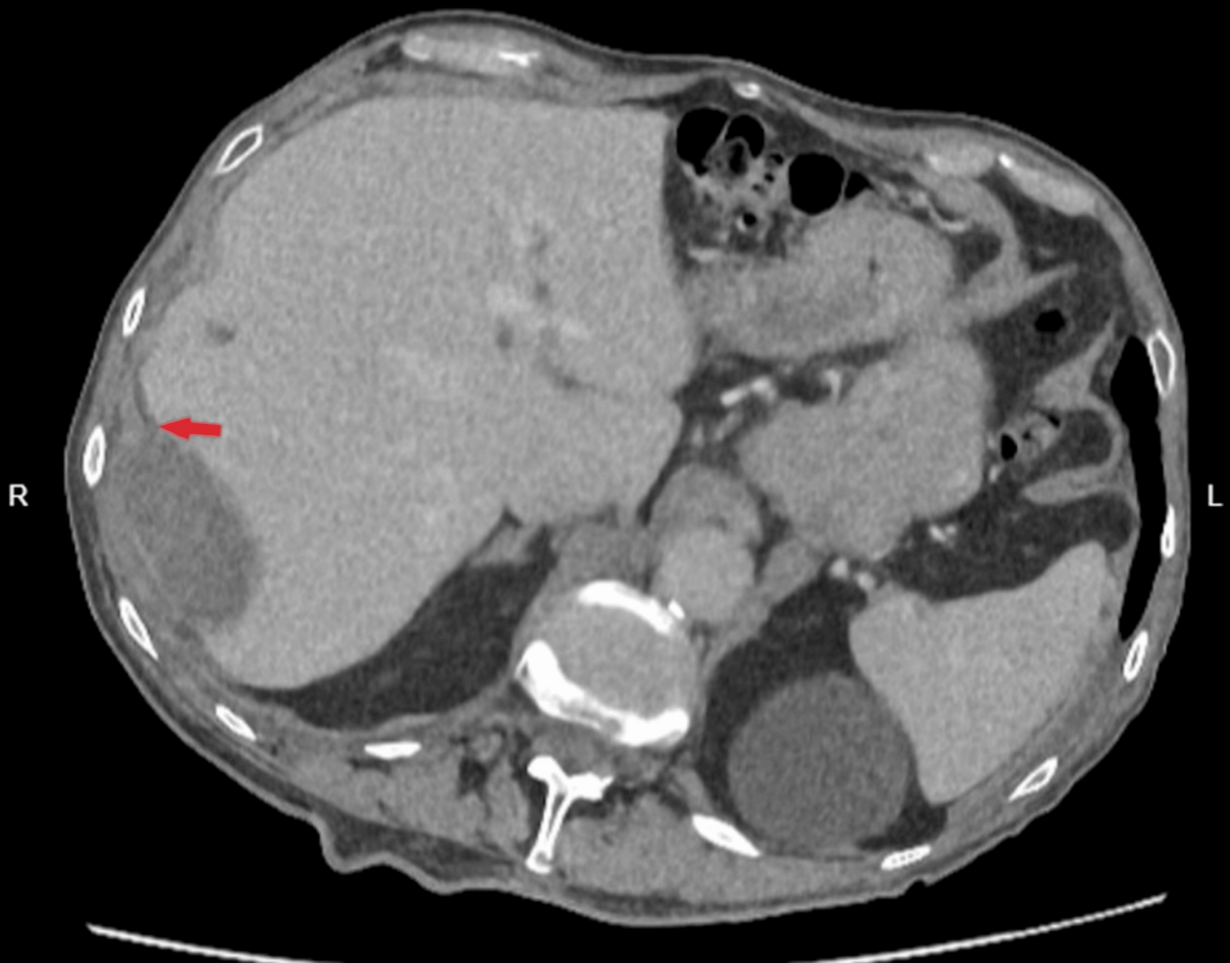
Contrast-enhanced CT abdomen and pelvis with contrast on Day 107 (postdischarge) showing regressing hematoma A small area of residual low attenuation is seen within the liver at the site of the previous drain. The remaining abscess cavities have completely resolved. The hematoma continues to shrink CT: computed tomography

During his prolonged admission of 107 days, the patient also experienced dysphagia. A barium swallow revealed aspiration and a distal esophageal filling defect, but a subsequent esophagogastroduodenoscopy and CT of the neck ruled out any underlying malignancy or structural abnormality. Following the TAE and optimization of his antimicrobial therapy, the patient stabilized. Serial imaging demonstrated a gradual, progressive reduction in both the multiloculated abscess cavities and the subcapsular hematoma. After completing an extended, microbiologically guided course of antibiotics, he showed significant functional improvement. He was eventually discharged to a hospital-at-home care program with a structured outpatient follow-up plan. Repeat imaging prior to discharge confirmed the near-complete resolution of the hepatic abscesses and the organizing hematoma (Figure 3).

## Discussion

This case illustrates several critical challenges in the contemporary management of PLAs, speciﬁcally addressing drainage failure, complex polymicrobial resistance, and life-threatening hemorrhagic complications. Percutaneous catheter drainage combined with systemic antibiotics is the cornerstone of PLA management. However, multiloculated abscesses, such as the one seen in this patient, are recognized as a significant predictor of percutaneous drainage failure [3].

The internal septations prevent adequate fluid evacuation, often necessitating the placement of multiple drains, the use of larger-bore catheters, or the instillation of ﬁbrinolytics. In this case, the complex architecture of the abscess contributed to the inadequate source of control and subsequent disease progression.

The microbiological proﬁle of this abscess was notably severe. While *Klebsiella pneumoniae *and *Escherichia coli* are the most common pathogens implicated in PLA, the isolation of *P. aeruginosa*, VRE, and Candida species indicates a highly vulnerable host and a complex, hospital-acquired or highly resistant infection profile [5]. Enterococcal liver abscesses, particularly those involving VRE, are associated with higher rates of treatment failure and mortality [6]. Furthermore, the presence of fungal organisms like Candida in a liver abscess is rare in nonneutropenic patients and is typically associated with severe systemic illness and poor outcomes [7]. The use of 16S rRNA sequencing in this case was pivotal, as it enhances diagnostic yield in culture-negative or complex polymicrobial infections, allowing precise tailoring of antimicrobial therapy [8]. The subsequent development of hepatotoxicity necessitated the cessation of tigecycline, highlighting the delicate balance required when managing multidrug-resistant infections with broad-spectrum agents in patients with compromised hepatic function [9]. The most dramatic aspect of this case was the spontaneous rupture of the PLA, resulting in a massive subcapsular hematoma and active arterial hemorrhage. While spontaneous rupture of a PLA into the peritoneal or pleural cavity occurs in a small percentage of cases, rupture leading to a subcapsular hematoma with active bleeding is exceedingly rare [4].

In the setting of spontaneous hepatic hemorrhage, TAE has emerged as a highly eﬀective, minimally invasive alternative to emergency laparotomy. Recent literature strongly supports the use of TAE as a ﬁrst-line intervention for achieving rapid hemostasis in patients with bleeding hepatic lesions, particularly in elderly or hemodynamically unstable patients who carry a prohibitive surgical risk [10,11]. In our patient, TAE successfully arrested the active extravasation, stabilizing the patient and allowing for continued conservative management of the hematoma and the underlying infection.

## Conclusions

Spontaneous rupture and hemorrhage of a PLA is a rare, catastrophic complication that requires a high index of suspicion, especially in patients who deteriorate despite appropriate initial drainage and antimicrobial therapy. This case highlights the limitations of percutaneous drainage in multiloculated abscesses and the growing challenge of multidrug-resistant polymicrobial infections. Crucially, it demonstrates the life-saving role of IR; TAE provides a rapid, safe, and deﬁnitive treatment for hepatic arterial hemorrhage, avoiding the signiﬁcant morbidity and mortality associated with emergency surgical intervention in critically ill patients.

## References

[1] Lam JC, Stokes W (2023). Management of pyogenic liver abscesses: contemporary strategies and challenges. J Clin Gastroenterol.

[2] Trillos-Almanza MC, Restrepo Gutierrez JC (2021). How to manage: liver abscess. Frontline Gastroenterol.

[3] Haider SJ, Tarulli M, McNulty NJ, Hoffer EK (2017). Liver abscesses: factors that influence outcome of percutaneous drainage. AJR Am J Roentgenol.

[4] Chen J, Zhao Z, Huang K (2025). Case report: massive subcapsular hepatic hematoma after spontaneous rupture of pyogenic liver abscess in a healthy young woman. Front Med (Lausanne).

[5] Sharma S, Ahuja V (2021). Liver abscess: complications and treatment. Clin Liver Dis (Hoboken).

[6] Oliosi E, Rossi G, Nguyen Y (2023). Enterococcal pyogenic liver abscesses: high risk of treatment failure and mortality. Eur J Clin Microbiol Infect Dis.

[7] Canouï E, Rossi G, Nguyen Y (2023). Analysis of 15 cases from a monocentric cohort of 307 liver abscesses. Mycoses.

[8] Song YG, Shim SG, Kim KM (2014). Profiling of the bacteria responsible for pyogenic liver abscess by 16S rRNA gene pyrosequencing. J Microbiol.

[9] Wei C, Liu Y, Jiang A, Wu B (2022). A pharmacovigilance study of the association between tetracyclines and hepatotoxicity based on Food and Drug Administration adverse event reporting system data. Int J Clin Pharm.

[10] Srinivasa S, Lee WG, Aldameh A, Koea JB (2015). Spontaneous hepatic haemorrhage: a review of pathogenesis, aetiology and treatment. HPB (Oxford).

[11] Onishi Y, Shimizu H, Oka S (2023). Transcatheter arterial embolization for subcapsular hematoma of the liver. Abdom Radiol (NY).

